# Distribution of breast cancer risk factors in two groups of healthy people referral to cancer registry and Shahid Mottahari center

**DOI:** 10.1016/j.dib.2018.05.045

**Published:** 2018-05-12

**Authors:** Rahmatollah Moradzadeh, Parvaneh Golmohammadi, Mohammad Hassan Kazemi-Galougahi, Majid Sartipi, Hooshmand Sharfi, Mohammad Ahmadpour

**Affiliations:** a Department of Epidemiology, School of Health, Arak University of Medical Sciences, Arak, Iran; b School of Health, Arak University of Medical Sciences, Arak, Iran; c Department of Epidemiology & Biostatistics, School of Public Health, Tehran University of Medical Sciences, Tehran, Iran; d Department of Epidemiology and Biostatistics, School of Health, Zahedan University of Medical Sciences, Zahedan, Iran; e Students Research Committee, Kermanshah University of Medical Sciences, Kermanshah, Iran; f Department of Public Health, Maragheh University of Medical Sciences, Maragheh, Iran

**Keywords:** Breast cancer, Cancer registry, Risk factor, Iran

## Abstract

The aim of this study is evaluation of breast cancer risk factors distribution in two groups of healthy people referral to cancer registry and Shahid Mottahari center in Iran. This study is cross-sectional study which is part of the study to estimate Gene-Environment Interaction in women with breast cancer with case-control studies in Shiraz. In this study, two control groups have been used. The sample size of 300 was specified for each group. Selection sources of groups include Cancer Registry Center and referred people to surgical and internal ward of Shahid Mottahari Clinic. Information collect tools have included Form No. 1 in Cancer Registry Center which includes information of age, use of oral contraceptives history, breastfeeding history, number of live births, age at menarche, age at first childbirth, etc. Considering the results obtained, it was showed that the highest frequency (144) in the group of Cancer Registry Center belongs to high school education, but the highest frequency of Shahid Mottahari Clinic is related to primary education (176).There is statistically significant difference between the two groups in terms of education, history of breast cancer in first-degree relatives and age at first birth, (*P*<0.05). Due to the easy availability of data on non-cancer patients referred to the cancer registry center, researchers may be encouraged to use them as a control group, but we must bear in mind that, this Group may be different in terms of some variables, and this difference leads to bias in the estimation of considered exposure effects.

**Specifications Table**

**Table:** 

Subject area	Medicine
More specific subject area	Public health
Type of data	Tables
How data was acquired	In this cross-sectional study, the women with breast cancer in shiraz as case group and two control groups have been used (Each group consisted of 300 people). Selection sources of groups include cancer registry center and referred people to surgical and internal ward of Shahid Mottahari Clinic. Information collect tools have included Form No. 1 in cancer registry center which includes information of age, use of oral contraceptives history, breastfeeding history, number of live births, age at menarche, age at first childbirth, etc.
Data format	Raw, analyzed
Experimental factors	In this study, two control groups have been used.
Experimental features	Statistical analysis has been performed using STATA 8.0 statistical software.
Data source location	Shiraz city, Iran
Data accessibility	Data are included in this article

**Value of the data**

• Breast cancer is one of the most commonly diagnosed cancer and a leading cause of cancer death in women in the world [1], [2], [3], [4], [5]. Therefore, further study is needed in this regard. The data from this study are in line with this overall goal.
• In some parts of Iran, the incidence of breast cancer is higher than in other places [6], [7], [8], [9], [10]. Therefore, collecting data about these cases and analyzing the risk factors of this type of cancer is very useful in preventing it. The data from this study are in line with this overall goal.
• In this study, two control groups have been used. Therefore, it can be said that the data obtained from this study is valuable.
• The data of this study show that there is statistically significant difference between the two groups (case and control) in terms of education, history of breast cancer in first-degree relatives and age at first birth.

## 1 Data

Considering the results obtained, it was showed that the highest frequency (144) in the group of Cancer Registry Center belongs to high school education, and the lowest frequency associated with illiterate group. But the highest frequency of Shahid Mottahari Clinic is related to primary education (176) and the lowest one is related to cases with academic education [11]. The results also showed a statistically significant difference between the two groups according to level of education (*P*=0.001). But in terms of history of live births and breastfeeding, there is no significant statistical difference between the two groups. But in terms of family history of breast cancer in first-degree relatives they had a significant difference that, the highest frequency of this variable (49) was in the group of breast registry center s and 17 cases were in the control group of Shahid Mottahari clinics. There was a statistically significant difference (*p*=0.001) between the two groups in terms of age at first birth that, the highest frequency (256) was associated with Shahid Mottahari clinic group with age at first birth 25–34 years. No significant difference was observed in terms of duration of contraceptive pills (ocp). And the statistical test can not be performed with the variable age at first menstrual, because the changes within both groups is constant. The above results are shown in Table 1.

**Table 1:** Distribution of frequency of study covariates among study population.

**Components**	**Shahid Mottahari clinic**	**Cancer registry**	***P***
Education			
Illiterate	30	64	0.001
Elementary education	120	176	
High school education	144	74	
College education	32	18	

History of breast feeding			
No	17	15	0.4
Yes	310	318	

History of parity			
No	0	1	0.49
Yes	291	286	

Family history of breast cancer			
No	49	17	0.001
Yes	278	316	

Age at first pregnancy			
Lower than 25	33	44	0.001
25–34	225	256	
35 and over or No pregnancy	60	25	

Oral contraceptive duration			
Minimum	0	0	0.78
Maximum	268	648	

Age at menarche			
Lower than 12	0	0	–
Over 12	268	291	

## 2 Study design, materials and methods

The research is part of the study to estimate Gene-Environment Interaction in women with breast cancer with case-control studies, and the only one in Shiraz. In this study, two control groups were used: 1. Women without breast cancer selected among women referred to Cancer Registry Center for reasons other than breast cancer (such as obtaining the necessary training in the field of prevention). Mammography diagnostic test had been done to these people. The center׳s clients include families of patients, people referred from private clinics or health centers in which educational and screening services are provided to referring patients. Most of these people have been settled in Shiraz and questionnaire was completed for them. In this study, available information were used as a control group for patients with breast cancer that is readily available. 2. Women referred to medical and surgical wards of Shahid Mottahari clinic were interviewed after consent to participate in the study. The questionnaire was completed for this group, like the first group, the control group of Cancer Registry Center was selected among cases referred to the Cancer Registry Center (Breast Clinic). Measured independent variables include the use of oral contraceptives, breastfeeding history, number of pregnancies, age at menarche, age at first childbirth.Sample size is determined using Quanto1.2 application (January 2007) [12]. That was obtained using substitution on the software with *α*=0.05 and power of 80% and calculated sample size of 293. In this study, 300 controls of surgical and internal wards of ShahidMottahari clinic and 300 controls from the cancer registry were used. Sampling was such that 300 cases were selected from the list of breast cancer patients in the cancer registry with a simple random sampling and their ages were specified. And among patients referred to the center that their breast cancer has not been confirmed, 300 subjects were selected by sampling with the age ±3 years for each case as an age-matched control. In addition, 300 persons of internal and surgical wards of ShahidMottahari clinic were selected with available (easy) sampling method by interview after the consent to participate in the study as a second control group matched for age ± 3 years.

Samples included people living in Shiraz city more than five years.

Information collection tool included Form No. 1 in Cancer Registry Center which includes information of age, use of oral contraceptives history, breastfeeding history, number of live births, age at menarche, age at first childbirth, etc. Statistical analysis has been performed using STATA 8.0 statistical software.
