# Supplementary material for: Distribution of breast cancer risk factors in two groups of healthy people referral to cancer registry and Shahid Mottahari center
Source: Data Brief. 2018 May 12;19:988–91. doi: 10.1016/j.dib.2018.05.045 (PMC5997904; doi:10.1016/j.dib.2018.05.045)
Supplement: Supplementary file 1 — Supplementary material [file mmc1.docx]

Conflict of Interest

All the authors confirm no conflict of Interest.
